# Current Applications of Diffusion Tensor Imaging and Tractography in Intracranial Tumor Resection

**DOI:** 10.3389/fonc.2019.00426

**Published:** 2019-05-29

**Authors:** Jamie D. Costabile, Elsa Alaswad, Shawn D’Souza, John A. Thompson, D. Ryan Ormond

**Affiliations:** Department of Neurosurgery, School of Medicine, University of Colorado, Aurora, CO, United States

**Keywords:** diffusion tensor imaging, tractography, glioma, resection, intracranial electrical stimulation

## Abstract

In the treatment of brain tumors, surgical intervention remains a common and effective therapeutic option. Recent advances in neuroimaging have provided neurosurgeons with new tools to overcome the challenge of differentiating healthy tissue from tumor-infiltrated tissue, with the aim of increasing the likelihood of maximizing the extent of resection volume while minimizing injury to functionally important regions. Novel applications of diffusion tensor imaging (DTI), and DTI-derived tractography (DDT) have demonstrated that preoperative, non-invasive mapping of eloquent cortical regions and functionally relevant white matter tracts (WMT) is critical during surgical planning to reduce postoperative deficits, which can decrease quality of life and overall survival. In this review, we summarize the latest developments of applying DTI and tractography in the context of resective surgery and highlight its utility within each stage of the neurosurgical workflow: preoperative planning and intraoperative management to improve postoperative outcomes.

## Introduction

Surgical resection remains the front-line treatment for many forms of intracranial tumor (1). Evidence from multiple large case series clearly supports the benefit of greater extent of resection (EOR) for patients with low-grade, high-grade, and recurrent gliomas with respect to their overall survival and progression-free survival outcomes (2–8). Moreover, preserving cortical and subcortical function is important for better postoperative outcomes and improved quality of life (9). Recent advances in neurosurgical planning and navigation technology seek to address two main issues: (a) non-invasive means of identifying tumor pathology and (b) non-invasive mapping of functional brain areas. Currently, the gold standard for each issue involves invasive methods such as biopsy and direct electrical stimulation (DES), respectively.

Surgical planning has benefited greatly from advances in neuroimaging. Yet, there remain a number of barriers preventing accurate determination of tumor pathology using noninvasive means alone. Biopsy is typically relied upon to determine pathology and grading. However, it is subject to sampling error (10, 11) and is often limited to easily accessible, enhancing regions of the tumor (12). More importantly, unnecessary surgical intervention poses a risk to patient comfort and quality of life, and risks complications especially when performed in eloquent regions of the brain (13). While DES is currently the most trusted method to identify functional tissue, depending on the geometry and grid arrangement of the electrode array, this technique has 2–3 mm or greater spatial resolution (14). As a result, DES is often accompanied with a margin of safety so that functional areas have a higher probability to be preserved. Yet, it does not always provide reliable responses and can potentially induce seizures. DES can also cause anxiety and fatigue for patients who must remain awake for extended periods of time for mapping during tumor excision.

Recent case series have highlighted the use of DTI to circumvent the weaknesses of both DES and biopsy. DTI measures water diffusion properties of neural tissue and can be used to approximate functionally relevant anatomical WMT in the brain. Within the context of intracranial tumor diagnosis and treatment, DTI has been used for planning neurosurgical interventions (15, 16), defining areas to excise and areas to avoid (17, 18), preoperatively differentiating tumor pathology, (19, 20), intraoperative estimation of brain shift (21), improving location/orientation targeting for DES sites (15, 22), improving postoperative functionality scores (23), assessing postoperative morbidity and mortality using preoperative information (24–26), and so on. Despite the diverse clinical applications for DTI within neurosurgical oncology, the method remains underutilized and lacks Level I evidence support. In resective surgery, DDT is primarily used in preoperative surgical planning and as an adjunct for the intraoperative functional information provided by DES. In the latter usage, tractography plays a limited role—providing location estimates for DES stimulation or as a coarse substitute for functional brain areas when DES is unavailable or not working properly.

In this review, we will discuss applications of DTI and DDT in the context of neurosurgical tumor resection, provide an in-depth description of current developments, and consider their limitations.

## Preoperative Applications of DTI

### Tumor Pathology Classification

Treatment strategies for intracranial tumors differ considerably based on pathology and grading, and optimal prognosis is dependent on strategy (27, 28). Non-invasive methods for diagnosis, such as conventional magnetic resonance imaging (MRI), currently do not provide the accuracy required for clinical decision-making (29). Gliomas are known to be a heterogeneous group with highly variable imaging qualities—attributed to variation in genetic and epigenetic mutations occurring early in tumorigenesis (30). To make matters more difficult, gliomas, and metastases can be difficult to visually differentiate, as these tumors display similar signal intensities and contrast enhancement patterns (31). While conventional MRI demonstrates some ability to classify pathology, resulting in a correct basic diagnosis 81% of the time (32), additional information is necessary to be clinically useful.

Diffusion coefficients in neoplastic brain tissue provide additional information for detecting tissue differences in and proximal to the tumor (33). Water movement in the brain is a blend of locally unrestricted, uniform fluid movement (isotropic component) within regions like the ventricles and restricted fluid movement (anisotropic component) within axonal membranes, myelin sheaths, and the extracellular matrix. At each voxel, an ellipsoid is calculated to approximate diffusivity in three-dimensional space, and each dimension (or aggregate of dimensions) may relay information about tissue properties or types (34, 35). Links between diffusion and cellularity, cell density, and proliferation have been reported: mean diffusivity (MD; average diffusion across spatial dimensions) decreases with increasing cellularity (29) and fractional anisotropy (FA; measure of directionality of diffusion in a given voxel) increases with increasing cell density and proliferation (36). It is important to note that these relationships are often specific to the pathology under investigation. Studies have shown that tumor pathology alters the diffusion properties of neural tissue in distinct ways (37–39). For instance, restricted water diffusion in solid tumors has been reported by many groups (36, 40).

Numerous studies have investigated the possibility of differentiating between gliomas and metastases (39, 41–52) and between low- and high-grade gliomas (53, 54) with mixed success. The lack of consensus may well be due to differences in DTI acquisition, preprocessing and postprocessing protocols, region of interest (ROI) location selection, and sample size. One commonality these articles share is applying logistic regression or receiver-operator curve analysis to classify pathology from ROI-derived TI coefficient averages, in or around the tumor (e.g., enhancing or immediate peritumoral regions) (49). Ultimately, two diffusion trends between high-grade gliomas and metastases were uncovered: increased FA intratumorally and peritumorally in gliomas and increased MD peritumorally in metastases (Table 1).

**Table 1:** Comparisons of diffusion coefficients in high-grade glioma(HGG) and metastasesfrom ROIs of enhancing and immediate peritumoral volumes.

	**FA**	**MD**	**CL**	**CP**	**cs**	**References**
High grade glioma vs metastases	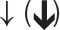	↑(↑)	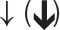	↑(↑)	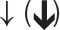	(41)
	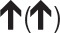	↑(↓)	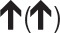	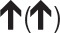	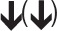	(45)
	↑(↑)	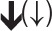	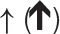	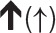	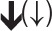	(46)
	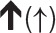	↓(↓)	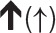	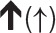	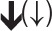	(47)
	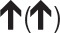	↓(↓)	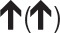	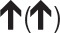	–	(48)
	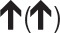	↓ (–)	–	–	–	(49)
	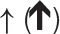	↑(↓)	–	–	–	(50)
	↑(↑)	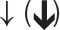	–	–	–	(51)
	↑(↑)	↑(↓)	–	–	–	(52)
	↑(↑)	↑(↓)	–	–	–	(42)
	– (↑)	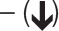	–	–	–	(39)
	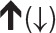	–	–	–	–	(43)
	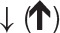	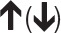	–	–	–	(44)
	↓(↑)	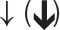	–	–	–	(53)

*Legend: ↑, increased in HGG compared to metastases; ↓, decreased in HGG compared to metastases; 0, no discernable difference; -, not reported/tested. Bold indicates significance in data, otherwise indicates trends in data. Arrows within parentheses indicate measurement from peritumoral volume, otherwise contrast-enhancing (bulk tumor) volume. Diffusion coefficients listed: FA, fractional anisotropy; MD, mean diffusivity; CL, linear anisotropy; CP, planar anisotropy; and CS, spherical anisotropy*.

Advanced techniques, including additional imaging modalities and/or more sophisticated machine learning methods, such as support vector machines and neural networks, may result in improved diagnostic performance (50, 55). For example, Chen *et al*. (55) applied a Bayesian network model incorporating DTI, T1-weighted, FLAIR, and MR perfusion measurements to differentiate between glioblastomas and solitary brain metastases. The predictive accuracy of the model was 0.94 with sensitivity = 0.96 and specificity = 0.92. For comparison, the specificity and sensitivity of those listed in Table 1 range between (0.77, 1.0) and (0.63, 0.92), respectively. Moreover, the research demonstrated 8 of 36 biomarkers retained its predictive power: 6 from DTI, 2 from MR perfusion, and 1 from FLAIR. Not only does this show the utility of the information contained in diffusion coefficients, it also demonstrates that combining imaging modalities with an advanced classification model may hold the key to accurate non-invasive histopathology.

### Presurgical Planning

Incorporating tractography in presurgical planning is beneficial for both neurosurgeon and patient as it improves defining the boundaries between eloquent areas and tumor, helping to formulate a patient-specific approach, thereby reducing the risk of postoperative neurological deficits. Studies have shown tractography aids in selecting the optimal surgical approach for corticectomy by comparing how potential access routes would impact WMT (56, 57). In planning the surgical approach, DDT provides the neurosurgeon with tumor proximity to WMT (58, 59), tumor impact on WMT (60), and extent of tumor invasion into the surrounding tissue (61, 62). Another improvement in surgical planning using DTI is the process of defining the resection boundary. Defining a resection boundary is a balance between maximally excising the lesion and preserving functional tissue. Here, altered WMT position and/or direction reflects displacement via tumor and decreased WMT density corresponds with vasogenic edema, tumor projections, or WMT destruction (63). DTI-defined tractography has also shown value in selecting patients for resective surgery, for example, removing patients with tumors found to be embedded in or in close proximity to functionally important regions (64). Furthermore, as methods and analyses continue to improve, DTI and DDT can aid in benefiting patients who are unable to undergo awake mapping and instead have surgeries under general anesthesia (21, 65).

### Extent of Resection (EOR) and Surgical Outcome Prediction

DTI and DDT show promise in preoperatively predicting the EOR, giving patients a more informed prognosis of their surgical outcome. Aggressive resection of DTI-defined abnormalities demonstrated a greater chance of progression free and overall survival (66). In a different study, DDT showed that maintaining corticospinal tract (CST) integrity correlated with a higher chance of total EOR (67). A rating scale has also been introduced, accounting for several clinical and radiological factors to predict successful EOR. Ranging from 1 to 8, the scale analyzes tumor margins, imaging, symptoms, and volume, with values >4 indicative of successful EOR (68). DTI helped to validate this score and is an area of future study.

Preoperative and intraoperative DTI and DDT have been explored as a means to predict functional ability and recovery following tumor resection. Specifically, reconstructing eloquent pathways and indirectly measuring axonal health have produced schemes to predict the effects that resection can have on motor, language, and visual function. Postoperative motor deficits have been linked to lower FA averages and higher MD averages in the tumorous hemisphere CST (69), as well as distances ≤8 mm between tumor and CST (70). Preservation of arcuate fasciculus (AF) and superior longitudinal fasciculus (SLF) correlate to lack of long-term language dysfunction, while damage to these DTI-defined tracts showed short-term and potential long-term deficits (25). Also interhemispheric connectivity, determined preoperatively with DDT, has been regarded as a risk factor of surgery-related aphasia (71). Injury to optic radiation (OR) during surgery commonly results in postoperative visual field deficits (VFD) (72, 73). One study showed a linear relationship between VFD score and OR injury defined by tractography, which ultimately may be avoided through intraoperative visualization of the tract (74). Also, shorter distances between tumor and Meyer's loop (a subdivision of OR) has been linked to postoperative VFD (75, 76).

## Intraoperative Applications of DTI

### Intraoperative Use of Preoperative DT Images

Recent publications indicate that preoperative DDT may reduce postoperative neurological deficits through visualizing eloquent WMT intraoperatively. The general consensus in the literature concerning tumors near the CST is that DDT can aid in preserving functional tissue and results in positive post-surgical outcomes (15, 77, 78). However, validation may require a combination of methods such as assessing tract preservation when used in conjunction with another brain-mapping software, such as the NY Tract Finder. The NY Tract Finder is a 3D brain mapping method that electrically stimulates and identifies motor tracts, so that they can be preserved during surgery (79). Tumor resections in 40 patients were performed safely without damaging motor tracts or causing any postoperative neurological deficit using NY Tract Finder. DTI-defined CST has also been successfully used in other scenarios including assessment of CST projection from the lesioned motor cortex in children with unilateral spastic cerebral palsy (80). The authors reported DDT was able to identify the CST controlling the more pathologically affected hand and found to be consistent with transcranial magnetic stimulation (TMS) data.

Language tracts are particularly variable between patients, and thus they are difficult to precisely trace. When implemented in conjunction with navigated TMS (nTMS), DDT visualization during surgery aided in preserving speech for patients with tumors adjacent to the SLF (16, 81). Negwer et al. (81) found that when performing nTMS-based DDT, 76.0% of language fiber tracts, such as the SLF, were detected in the examined patients, as opposed to 39.9% when using the cubic ROI-based protocol. Out of 37 patients with left-sided perisylvian lesions, 22 patients (59.5%) received nTMS with 5 pulses of 5 Hz, 8 patients (21.6%) received 5 pulses of 7 Hz, and 7 patients (18.9%) received 7 pulses of 7 Hz. Compared with the nTMS-based DDT, the cubic ROI-based protocol displayed a better visualization of the AF (97.3% vs. 75.7%; *p* < 0.05) and of the SLF. The shorter language tracts, such as AF or the uncinate fascicle (UF), were visualized more effectively using the nTMS-based approach (*p* < 0.001); the UF was not detectable with the cubic ROIs-based protocol in either case. The fibers-to-tract ratio using nTMS-based DDT was 236 ± 73 and 286 ± 9 using the cubic ROIs-based protocol, which are comparable values and are both within the range Negwer *et al*. defined as ideal for a clear DDT result (fibers/tract 0–500) (81). The authors state that these results examining language-related DDT by nTMS still have to be confirmed by subcortical stimulation in future studies. This technique has also been used to aid in preserving motor function in patients with tumors adjacent to the CST in another study (82).

A “functional” approach based on preoperative fMRI and DTI scans in tandem with intraoperative neurophysiological monitoring shows promise as the current standard in the management of lesions in the dominant atrium (DA), a crossroad of eloquent WMT difficult to preserve with a standard “anatomical” approach (83). In a study consisting of asleep surgery in 43 patients, the combination of fMRI and DDT identified locations of the AF, inferior fronto-occipital fasciculus (IFOF), OR, and CST proximal to the DA resulting in gross total resections (< 2mm^3^ residual tumor) in 93.0% of patients and few postoperative deficits (9 transient, 3 permanent cases) (84).

### Intraoperative DTI (IDTI)

Brain shift is known to affect the reliability of preoperatively generated DDT (85, 86). This hurdle can be reduced by updating tractography through intraoperative imaging. Image processing and fiber tract reconstruction can be completed in under 20 minutes (hence it is feasible to carry out DTI and DDT during surgery) and then integrated into navigational datasets (21, 87, 88). Marongiu et al. (88) directly compared postoperative outcomes of supratentorial glioblastoma resections accompanied by iDTI to those without accompaniment (due to MR equipment outage). For surgeries with iDTI, EOR was markedly higher (88.5% vs. 44%) and six-month progression free survival was greater as well (73.1% vs. 38.9%). Another benefit of increasing the reliability of tractography via iDTI, is performing more asleep surgeries. Research has shown an average of >95% resection in asleep resection near speech and language areas is possible using a combination of iDTI and fMRI (21).

That said, intraoperative imaging requires additional time and surgical preparation (89). A method to circumvent additional resources is by incorporating models compensating for intraoperative brain shift. Miga et al. (90) created a sparse-data-driven biomechanical model to predict physiological brain shift by accounting for typical deformation-inducing events such as cerebrospinal fluid drainage, hyperosmotic drugs, swelling, retraction, resection, and tumor cavity collapse. Their results are encouraging and await further comparisons that include: (1) detailed intraoperative MR validation, (2) workflow analysis, and (3) optimal forms of data visualization. Alternatively, Morin et al. (91) proposed an intraoperative ultrasound technique where a navigated ultrasound acquisition is performed directly in contact with the organ and doppler and B-mode images are recorded simultaneously, enabling the extraction of the blood vessels and probe footprint. Then, a constraint-based simulation registers the pre- and intraoperative vascular trees along with the cortical surface with the probe footprint. Finally, preoperative images are updated to provide the surgeon with current images of brain shape to navigate during resection.

### Combining DDT and DES

Direct electrical stimulation (DES) is regarded as the gold standard for mapping brain function, though evidence suggests improvements are needed. While DES provides real-time, approximate spatial information about eloquent fibers—enhancing surgical performance and safety—it is time consuming and places the patient at risk for intraoperative seizures due to repeated direct electrical stimulation (92, 93). Moreover, DES has been shown to induce hemodynamic changes across large areas (77 sq. mm to 350 sq. mm) and the effect is difficult to predict as it depends on many local and remote physiological and morphological factors (94). It is misleading to assume DES allows us to draw unequivocal conclusions about the role of stimulated brain areas.

**Figure 1:**
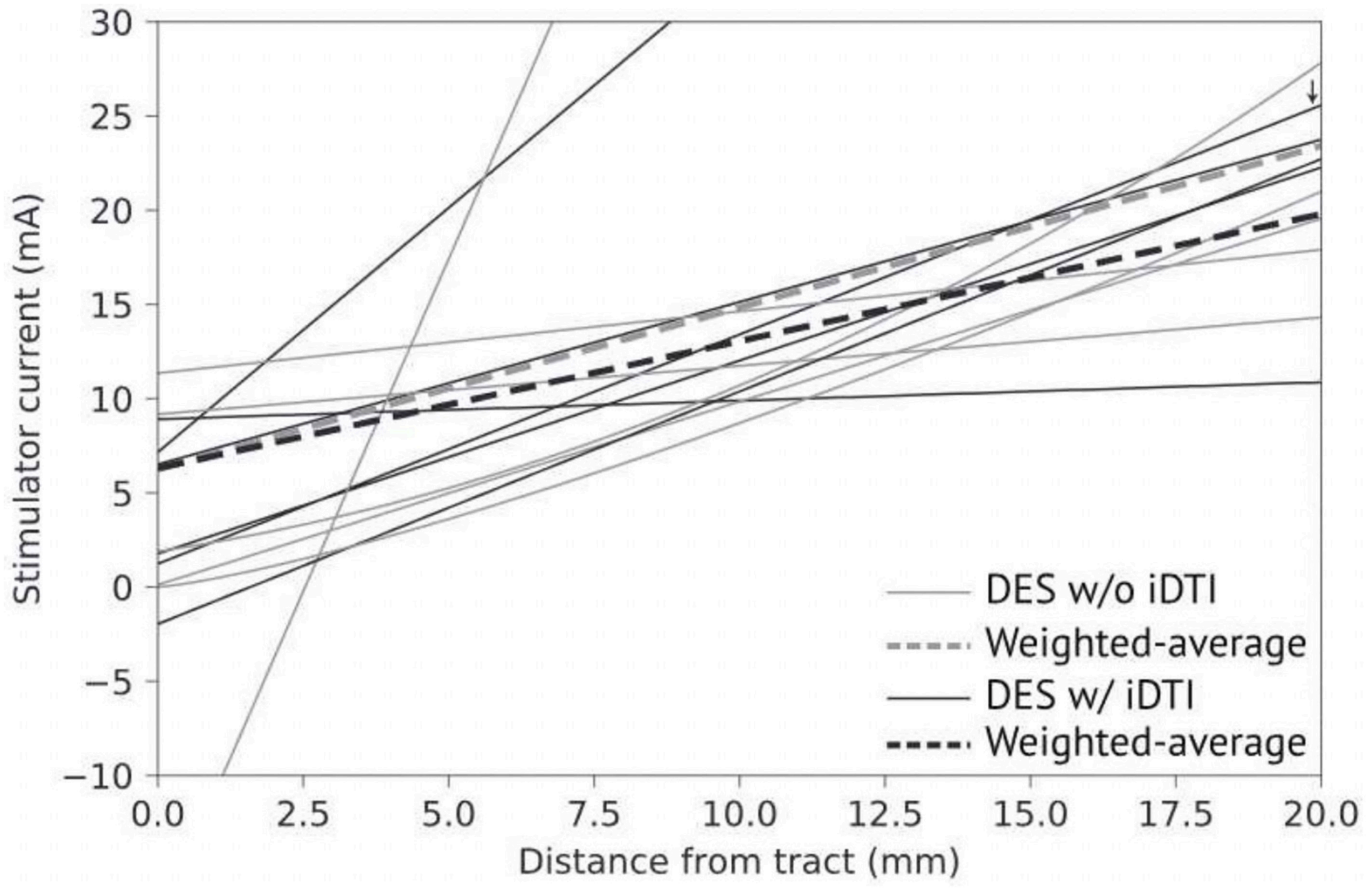
Comparison of DES current to distancefrom DDTfrom imaging performed intraoperatively (black) or pre/postoperatively (gray). Averages weighted by number of stimulation points divided by number of patients per study for both imaging methodologies (dash-lines). One publication studied the optic radiation (small arrow), all others studied the CST.

A collection of studies have demonstrated a direct relationship between DES current magnitude and the distance from probe to DTI-defined tracts; as the proximity to tracts increases, stimulation current decreases (22, 95–99). This relationship becomes more reliable when tract locations are updated via intraoperative imaging or ultrasonography (22, 86, 100–103). Figure 1 illustrates the general clustering of published DES current to DTI-defined tract distance correlations with respect to intraoperatively acquired images. Until verified models relating current-to-distance, the gold standard for mapping functionality may not be DES alone but DTI combined with DES. Combining these methods has been shown to be more effective at delineating motor pathways, yielding mean EOR values of 97.2 and 97.7% of cerebral glioma in eloquent areas (26, 100).

Further support for the efficacy of combining tractography and electrical stimulation (ES) was demonstrated in a study by Yamao et al. (104) evaluating cortico-cortical evoked potentials (CCEP) as a new intraoperative monitoring method of the dorsal language pathway. The preoperative AF tract can be dislocated or interrupted due to brain edema or infiltration of the tumor; tools like CCEP address this issue with the aid of preoperative fMRI and probabilistic tractography. When high-frequency ES to the floor of the resection cavity elicited language impairment (4 out of 21 patients), the distance between the subcortical stimulus site and the AF tract was within 5 mm. This illustrates DTT's value as a sophisticated neurosurgical approach for preserving language function. However, according to the authors, further studies are needed to establish a solid cut-off value to develop the CCEP monitoring as an efficient intraoperative method for preservation of the dorsal language pathway.

## Improvements to DTI and DDT

Although DTI and DTT present many advantages and have demonstrated utility in approximating the geometry of WMT in the context of neurosurgical interventions, it is limited in its ability to resolve multiple fibers and is susceptible to partial volume effects (105). Progress in addressing these issues has been made by using alternative models to calculate diffusion properties, more complex image sequences acquired from additional magnetic field gradient directions and magnitudes, or a combination of the two.

Alternative models involve expanding the diffusion model to account for higher order diffusion features [e.g., diffusion kurtosis imaging (DKI)], substituting the tensor with another shape [e.g., constrained spherical convolution (CSD)], or modeling the Fourier transform relationship between the diffusion MR signals and the underlying diffusion displacement [e.g., generalized Q-ball imaging (GQI)]. While models like DKI and CSD require more extensive image sets, improvements to DDT are possible by applying models other than DTI. For example, Zhang et al. (106) demonstrated CSTs passing through areas of edema could be visualized with the GQI model but not DTI (106).

Advanced diffusion imaging sequences include diffusion spectrum imaging (DSI), high angular resolution diffusion imaging (HARDI), DKI, and Crossing Fiber Angular Resolution Intra-voxel structure (CFARI). Tractography based on DSI has the capacity to image crossing fibers in neural tissue using probability density functions, which specify microscopic displacements of MR-visible spins using 3D Fourier encoding of displacements. Unlike DTI, DSI is able to resolve crossing fibers, accurately reconstruct confluence of pathways, and demonstrate the decussation of fibers in the optic chiasm (107). In contrast, HARDI constructs maps approximate the diffusion orientation density functions. Caverzasi et al. (25) demonstrated that combining HARDI and Q-ball imaging improved the resolution of tractography in regions with crossing fiber populations, presenting more complex structures than DDT. During a study of 35 patients with gliomas, they found that preservation of the left AF and the temporoparietal component of the SLF (SLF-tp) was consistent in patients without language deficits at the long-term follow-up after glioma resection. The AF and SLF-tp were affected in patients with short-term language deficit, and predictive of long-term deficits. The authors concluded that the lack of postoperative deficits supports intraoperative use of language tracts. More advanced image sequences that calculate diffusion magnitudes and directions involving complex distributions of intravoxel fiber orientation are available. Across the board, these techniques are based on improving resolution by acquiring more images from more magnetic field gradient directions.

Overall, novel applications of DTI and DDT have demonstrated that preoperative, non-invasive mapping of eloquent cortical regions and functionally relevant WMT and their use with other intraoperative adjuncts like DES and nTMS help to reduce postoperative deficits, improving quality of life and overall survival in glioma patients. Future more advanced imaging sequences will continue to improve the utility of WMT depiction in neurosurgery, improving functional outcomes in glioma patients.

## Author Contributions

JC devised the structure and focus of the article, wrote a significant portion of it, and managed the manuscript through its composition and publication processes. EA contributed to the publication by writing and editing portions of the intraoperative and limitations sections. SD contributed to the publication by writing and editing portions of the preoperative section. JT and DO were equally important to crafting the manuscript’s focus and editing of the article. JT and DO share senior authorship.

### Conflict of Interest Statement

The authors declare that the research was conducted in the absence of any commercial or financial relationships that could be construed as a potential conflict of interest.
